# Prevention of pediatric functional constipation: a narrative review

**DOI:** 10.1016/j.jped.2026.101531

**Published:** 2026-04-11

**Authors:** Mauro Batista de Morais

**Affiliations:** Universidade Federal de São Paulo, Escola Paulista de Medicina, Disciplina de Gastroenterologia Pediátrica, São Paulo, SP, Brazil

**Keywords:** Breastfeeding, Dietary fiber, Constipation, Infant, Child, Adolescent

## Abstract

**Objective:**

To conduct a narrative review of strategies and actions that can contribute to the prevention of functional constipation in children and adolescents.

**Source:**

This narrative review used articles indexed predominantly in the PubMed database and compiled by the author over the past 30 years.

**Summary of the findings:**

No specific prospective population-based studies have evaluated the effectiveness of preventive measures against constipation. However, some measures can contribute to the prevention of functional constipation, such as training healthcare professionals and educating the general population about the importance of breastfeeding, providing proper toilet training guidance, and encouraging actions to avoid postponing bowel movements. Nutrition is important at all life stages. When breastfeeding is prematurely discontinued, infant formulas are more effective than cow’s milk. Dietary fiber and water intake are frequently below recommended levels. Therefore, after introducing complementary feeding throughout life, the adoption of healthy eating habits, including adequate dietary fiber and fluid intake, should be recommended.

**Conclusions:**

Preventive measures for functional constipation have rarely been discussed in the literature. Training healthcare professionals and educating the general population are important for understanding the physiology of defecation from newborns to adulthood and avoiding unnecessary dietary changes or therapeutic measures. After introducing complementary feeding throughout life, the adoption of healthy eating habits, including adequate dietary fiber and fluid intake, should be recommended.

## Introduction

Constipation is a very common symptom that can occur at any age. It can appear acutely during an acute infectious disease, post-surgery, or even after a change of bathroom during travel, and is reversible after the triggering factor resolves without the need for prolonged treatment [1,3]. In contrast, constipation can be chronic and may be a consequence of various diseases, such as Hirschsprung's disease, celiac disease, hypothyroidism, opioid use, or even secondary to cow's milk allergy (CMA) [1., 2., 3., 4., 5., 6.]. In this context, a differential diagnosis is very important in pediatric patients with constipation [1., 2., 3., 4., 5., 6.]. It should be emphasized that the differential diagnosis between functional constipation and constipation secondary to CMA is a major challenge, especially in the first years of life [7,8]. Thus, in the ESPGHAN/NASPGHAN (European Society for Paediatric Gastroenterology, Hepatology and Nutrition/ North American Society for Pediatric Gastroenterology, Hepatology and Nutrition) guidelines [2] for the diagnosis and treatment of functional constipation, in the Rome IV criteria [8], and in other studies in the literature [7], there is a consensus that an elimination diet of cow milk proteins for diagnostic purposes should be considered in cases of functional constipation that do not respond to conventional treatment. In this situation, patients should follow the general approach adopted in food allergies; that is, they should undergo an oral food challenge to confirm the diagnosis after achieving normalization of bowel habits without the concomitant use of laxative medications during the elimination diet [9,10]. Discussions on the diagnosis, treatment, and prevention of food allergies, including CMA, have been revisited in recently published consensus statements [9,10].

Functional constipation accounts for over 95 % of constipation cases and is included in functional gastrointestinal disorders (disorders of the gut-brain interactions) [1., 2., 3., 4., 5., 6.,^11^]. Functional constipation is also the most prevalent functional gastrointestinal disorder in all age groups from 6 months of age, as evidenced in infants [12,13], children [14], adolescents [14], and adults [15]. The Rome criteria constitute the most widely accepted diagnostic criteria, requiring a minimum duration of 30 days of clinical manifestations to characterize the chronicity of the disease [8,11].

However, there are some concerns that the Rome criteria may be too restrictive for characterizing functional constipation [16,17], delaying diagnosis and treatment, which may be associated with a worse prognosis [2]. Functional constipation can cause biopsychosocial problems in addition to physical clinical manifestations [1., 2., 3., 4., 5., 6.].

Furthermore, functional constipation presents with a wide spectrum of severity, ranging from mild oligosymptomatic cases whose clinical manifestations are not always recognized [18] as severe cases that do not respond adequately to conventional treatment [1., 2., 3., 4., 5., 6.]. Discussing therapeutic options for patients with refractory constipation was beyond the scope of this study. It is worth remembering that early diagnosis and effective treatment of functional constipation are considered a factor associated with a better prognosis [2].

Moreover, functional constipation results in high costs for the public health system or for the family budget, including expenses for consultations, tests, and medications [3].

In childhood and adolescence, the basic principles of treating functional constipation have been well established since the 1990s [1,2,19., 20., 21., 22., 23.]. Whenever necessary, treatment must be initiated by successful emptying of impacted feces in the rectum and colon, followed by maintenance measures to recondition and normalize bowel function and prevent the recurrence of fecal impaction. Laxatives and educational measures are recommended for maintenance, including changes in lifestyle and eating habits [1,2,19., 20., 21., 22., 23.]. The most comprehensive and specific recommendations for the pediatric age group were developed by the NASPGHAN, the-ESPGHAN[2,20,21], and the NASPGHAN Neurogastroenterology and Motility Committee [24]. They also highlight measures aimed at controlling functional constipation that does not respond to treatment [2,24,25].

However, these guidelines do not include measures that could potentially prevent functional constipation. Defining preventive measures for functional constipation is crucial, considering its public health implications, physical and biopsychosocial suffering, and financial costs. Therefore, the objective of this article is to review the strategies and actions that can contribute to the prevention of functional constipation in children and adolescents.

## Methods

This narrative review included articles indexed predominantly in the PubMed database. Not only original articles but also review and recommendation articles, as well as articles compiled by the author over the past 30 years, were considered.

## Results

In the PubMed database, a search using the words "constipation and prevention" as title words and the filters "all children" and "adolescence" identified only six articles published between 1986 and 2017 (search on 12/27/2024) [26., 27., 28., 29., 30., 31.]. Despite specifying the word "prevention" in the title, three of these articles did not discuss the subject in depth [27,29,31]. One article was a review on the role of water intake, that has been used in one of the topics of this article [31]. Another was a clinical trial that showed that the administration of a certain probiotic from the neonatal period to 90 days of life led to a statistically significant increase in bowel movement frequency at the end of the follow-up period [28]. However, a specific definition of constipation was not used [28]. Finally, the experience reported almost 40 years ago by Rappaport and Levine [26] provided an excellent description of the opportunities for preventing functional constipation, focusing on actions to be adopted throughout the childhood development process, as discussed below [26].

### Prevention of functional constipation in the context of childhood development and the biopsychosocial environment

In 1986, Rappaport and Levine published an excellent article focusing on the prevention of constipation, integrated with the stages of childhood development [26]. This article emphasizes that parental guidance and education regarding changes in bowel function throughout the first years of life are important strategies for preventing constipation and other bowel-related disorders [26].

Therefore, physicians should provide appropriate parental guidance to foster confidence and competence in interpreting and understanding the normal evolution of a child’s bowel habits. This avoids unnecessary treatment and inappropriate actions resulting from anxiety and fear generated by a lack of knowledge about normal variations in bowel physiology [26].

Generally, the fetus does not pass meconium before birth. Passage of meconium during pregnancy may be indicative of fetal distress. The first passage of meconium occurs within the first 24 h of life. Premature infants weighing < 1500 g at birth may have their first meconium passage after 48 h. Delayed meconium passage is an alarm signal that should be evaluated by a pediatrician [2,5,8,20,21]. After birth, infants are fed colostrum and breast milk, which cause changes in stool characteristics known as transitional stools. Thus, throughout the first few days of life, changes in stool characteristics are observed depending on the type of feeding. Exclusively breastfed infants have looser stools that are passed more frequently (approximately five times per day), whereas formula-fed infants have firmer stools with an average frequency of two bowel movements per day [32., 33., 34.].

In the first months of life, two conditions may occur that can cause concern in parents. The first is pseudo-constipation, which consists of the elimination of loose stool without pain or difficulty, with a prolonged interval between bowel movements (up to one or two weeks) [35]. Pseudo-constipation is not associated with excessive crying, abdominal distension, or vomiting. Pseudo-constipation should be considered a normal variation in bowel habits that occurs in approximately 5 % of breastfed infants and does not require treatment [31]. The second is infantile dyschezia, which can be seen up to 9 months of age and consists of at least 10 min of straining, crying, and facial flushing before the end of a bowel movement, in the absence of other health problems [1]. Infant dyschezia appears to be caused by a lack of coordination between abdominal muscle contraction and pelvic relaxation and represents a normal phase of childhood development [1]. Therefore, a lack of knowledge about pseudo-constipation and infantile dyschezia can lead to unnecessary and harmful parental behaviors, such as discontinuing breastfeeding or unnecessary changes to infant formula. Unnecessary use of suppositories and other anal stimulants should be avoided [8,26].

More complex motor and cognitive skills, such as walking and talking, appear by the end of the first year of life. At this stage, parental reports of stool retention behavior may emerge, generally beginning with episodes of painful bowel movements [26]. In this situation, the child develops stool retention maneuvers instead of allowing the natural sequence of the normal bowel movement mechanism. Retentive behavior is considered one of the main mechanisms linked to the onset and chronicity of constipation [1., 2., 3., 4., 5., 6.,^8^,^11^].

During the acquisition of anal sphincter control, the lack of a harmonious relationship between parents and children can facilitate the onset or worsening of retentive behaviors [26]. The recommendation is that painful bowel movements should be controlled before starting toilet training [3., 4., 5., 6.,^8^,^11^,^26^]. The age at which bowel and urination control are acquired varies across cultures. In Western countries, the age for acquiring anal sphincter control has increased from approximately 24 to 36 months [36]. There are several techniques for toilet training [6]. The child-oriented toilet training method is recommended by the American Academy of Pediatrics, the Canadian Society of Pediatrics [6], and the Brazilian Society of Pediatrics [36]. During toilet training, parents should be guided to positively support their children during the various stages of the process [6,36]. Training should generally begin between 18 and 24 months when the child shows signs of readiness, such as staying dry for a couple of hours at a time, showing interest in using the toilet, and being able to follow simple instructions [6,36]. The child should remain in an appropriate position on the potty or toilet with a seat reducer and footrest for periods of approximately 5 min. The gastrocolic reflex can be used during the postprandial period [6,36]. Punishment and immediate expectations should be avoided. Parents should be patient and offer positive reinforcement without exaggeration [6,36]. Imposing and coercive toilet training is classically considered one of the determining factors of withholding behaviors, which can be followed by fecal retention and functional constipation [1., 2., 3., 4., 5., 6.,^8^,^11^].

The pain-retention-pain cycle may begin or recur at school age, owing to phobias caused by a lack of cleanliness and privacy in school bathrooms. Other contributing factors include rigid school schedules and adherence to busy extracurricular activities [26].

Retentive behavior was identified in 70 % of school-age children with constipation in an epidemiological study, and was more frequent than other clinical manifestations, such as increased bowel movement intervals and the passage of stool that clogs the toilet. These data suggest that retentive behavior may trigger the onset of other clinical manifestations of functional constipation [18]. In patients treated at a specialized outpatient clinic, retentive behavior was found in 68 % of preschoolers, 41 % of schoolchildren, and 28 % of adolescents [37]. Therefore, recommending that retentive behavior be avoided by prioritizing defecation may represent a strategy to prevent the onset and progression of functional constipation.

Factors related to family dynamics and the social context can also influence the onset of functional constipation. Certain events can mark the onset of bowel movement disorders, such as the birth of a sibling, exposure to parental marital problems, moving to a new home or school, or the loss of a loved one. These events are milestones that require special attention from parents to address these adversities and prevent children from developing bowel movement disorders [1., 2., 3., 4., 5., 6.,^8^,^26^,^36^].

Exposure to violence and victimization in children and adolescents is associated with constipation and other gastrointestinal disorders [3., 4., 5., 6.,^8^,^11^]. A study conducted in Brazil using a comprehensive questionnaire revealed a high frequency of exposure and victimization to violence of varying degrees of severity among adolescents [38]. A greater number of questions indicated that violence was associated with a greater risk of functional constipation and irritable bowel syndrome [38]. Violence can influence the interaction between the brain and gut, facilitating the development of functional gastrointestinal disorders [3,4,6]. The possibility that maternal exposure to domestic violence is associated with a greater risk of constipation in children has also been discussed [39]. Therefore, protecting children and adolescents from exposure to violence and victimization can contribute to the maintenance of balanced mental and physical health, including bowel habits.

Other family factors may predispose patients to functional constipation. For decades, it has been emphasized that both children and other family members should have a healthy diet, prioritizing foods rich in dietary fiber and reducing foods rich in fats and simple carbohydrates [26]. In Brazil, an association has been observed between low dietary fiber consumption by mothers and children with constipation [40]. Therefore, it is often difficult for children to adhere to healthy eating habits when their parents and other family members do not do the same [26].

The role of genetic factors in the etiology of functional constipation can also be considered in the family context. A literature review highlights that no genes specifically linked to functional constipation have yet been identified; however, many genetic syndromes have constipation as one of the phenotypic manifestations [41]. In turn, a study of mono- and dizygotic twins has suggested that genetic factors can explain up to 59 % of the concomitant occurrences of constipation [42]. Other evidence compiled in the literature suggests an association between the fingerprint arch pattern and constipation. However, other studies, such as one conducted in Brazil, have not confirmed this finding [43]. In another Brazilian study, only slight agreement was observed between the presence of constipation in adolescents and their mothers or biological fathers, according to the Kappa coefficient (0.12 and 0.05, respectively) [44].

### Nutrition in the first year of life for the prevention of constipation

Approximately half of the children with severe functional constipation treated at specialized pediatric gastroenterology clinics experience disease onset in the first year of life [45., 46., 47.]. During this period, isolated or not gastrointestinal signs and symptoms may occur. These clinical manifestations may fulfill the Rome criteria for functional gastrointestinal disorders in infancy [8,12,13,48]. Regurgitation, colic, and dyschezia in infancy appear to be more closely related to the functional maturation of motility, digestion, and the establishment of intestinal microbiota [8,48]. These clinical manifestations generally disappear during the first year of life. However, functional constipation is more common in the second six months of life [12,13], and the type of nutrition is important for determining bowel habits.

Breastfeeding reduces infant mortality and the risk of developing chronic non-communicable diseases in adulthood [49]. In Brazil, breastfeeding rates have increased [50., 51., 52.] due to pediatricians’ actions and other public health initiatives [53].

The advantages of breastfeeding should be expanded to include the prevention of constipation. Table 1 presents the main results from articles [13,34,35,54., 55., 56., 57., 58., 59., 60., 61., 62., 63., 64., 65., 66., 67., 68., 69.] indexed in the PubMed database that linked breastfeeding and constipation.

**Table 1:** Summary of the original articles indexed in PubMed relating breastfeeding and constipation.

**Settings**	**Participants**	**Relation to breastfeeding**
Pediatric Gastroenterology Clinic São Paulo, Brasil Morais (1996) [54]	58 patients with functional constipation and 58 controls aged <12 years	History of exclusive breastfeeding duration: 1 and 3 months, respectively (*p* = 0.002).
Primary Care Unit Embu, São Paulo, Brasil Aguirre (2022) [35]	275 infants (25.1 % had constipation) aged <24 months	Artificial feeding was associated with higher chance of constipation (OR = 4,5; CI95 % 1,2;16,1) in relation to predominant breastfeeding.
Pediatric Clinic Italy Iacono (2005) [55]	2879 consecutive infants (16.6 % had constipation) aged <6 months attended by 150 Pediatricians	Infants with constipation were linked to a low frequency of breastfeeding (76.3 % versus 84.9 %; *p* = 0.007).
Well-child clinic Ankara, Turkey Tunc (2008) [56]	1021 children in the first two years of life	Hard stool was seen in only 1.1 % of exclusively breast-fed infants, while it was 9.2 % in formula-fed infants (*p* = 0.001). Stool frequency was igher (*p* = 0.0001) in exclusively breast-fed infants (3/day) in relation to breast-fed + formula-fed (1/day).
Primary Care Unit Osasco, São Paulo, Brasil Souza (2012) [57]	302 infants aged 6 to 24 months (22.2 % had constipation)	Partial breastfeeding and artificial feeding were associated with a 3.0 fold increased risk of developing constipation (*p* = 0.03).
Well-child clinic Ankara, Turkey Çamurdan (2014) [58]	125 infants followed from birth to 12 months	Median stool frequency per day was higher (*p* < 0,05) among the infants being on exclusive breastfeeding during the first 5 months of life compared to the infants being formula in addition to breastfed.
Day-care centers Seoul, Korea Park (2016) [59]	217 children (0.3 % had constipation) aged 25 to 84 months	History of breastfeeding for <6 months was more frequent (*p* = 0.033) in children with (94.1 %) constipation than in children without constipation (62.7 %).
Birth cohort study Bristol, UK Heron (2018) [60]	8435 participants of the Avon Longitudinal Study Prevalence of soiling (from 5.2 to 7.2 %) and constipation (from 9.5 to 14.5 %) between 4 and 9 years	There is little evidence of an association between breastfeeding duration and later problems with constipation and/or soiling,
Cohort followed-up at a clinic and in the community (First Baby Study) Pennsylvania, USA Pattisson (2019) [61]	2918 women were interviewed at 6-, 12-, 24-, and 36-months of their children’s age.	Overall, the findings support the hypothesis that duration of breastfeeding is associated with fewer reported episodes of diarrheal and/or constipation at 6, 12, and 24 months.
Cross-sectional, multicenter study at well-baby clinic Belgian, Italian, and Netherland Steutel (2020) [12]	1698 infants age 0–12 months (24.7 % had at least one FGID)	Formula feeding was associated (OR = 1.41 [1.01–1.98]; *p* = 0.045) with any FIGD in relation to breastfeeding
Cross-sectional study at a well-baby clinic Kuala Lumpur, Malaysia Chew (2020) [62]	534 healthy infants younger than 12 months of age (1.1 % had functional constipation)	Breastfeeding was only associated with reduced risk of infant regurgitation.
Pediatric Gastroenterology Clinic Ankara, Turkey; Agakisiyeva (2022) [63]	41 patients with functional constipation and 55 age-gender matched controls between ages 4–18 years	Breastfeeding > 18 months was more common in controls (*p* = 0.039).
School-based survey Sergipe, Brazil Oliveira (2021) [64]	1051 children (23 % had functional constipation) aged 2 to 6 years	Bottle feeding at 0–6 months of age increased the risk of functional constipation at preschool age (whole cow's milk: OR = 17.0 [95 % CI: 7.3–39.5]; infant formula: OR = 6.4 [95 %: CI 3.4–11.8], mixed breastfeeding: OR = 10.8 [95 % CI 4.6–25.7]
Cross-sectional study in a private pediatric clinic Brazil (all geographic regions) Morais (2022) [13]	4506 infants aged <12 months old (7.6 % with functional constipation)	OR = 1,18 (CI95 %: 0.86–1.41, *p* = 0.451) of mixed + artificial feeding in relation to exclusive breastfeeding
Cross-sectional study at governmental hospital and a governmental kindergarten. Hanoi, Vietnam Chia (2022) [65]	1511 subjects aged 0–48 months using Roma IV for FIGDs (Functional constipation: 1.5 % and 5.6 %, respectively, in subjects aged 0–12 months and 12–48 months).	Formula feeding initiation at 1 – 2 months was associated with functional constipation (OR = 18.6, 95 %; CI95 % = 1.6 –219.4)
Prospective observational birth-cohort study Gothenburg, Sweden Gatzinsky (2023) [34]	122 healthy full-term infants with information collected at 2 weeks and 2, 6, and 12 months of age.	Breastfeeding at 2 weeks of age decreased the odds (OR=0.16; CI95 %:0.04–0.68) of developing functional constipation
Japan Environment and Children’s Study cohort Japan (15 Regional Centers) Motoki (2023) [66]	70,078 mother-toddler pairs (11.6 % with functional constipation at 3 years of age)	Breastfeeding period duration of ≥ 7 months was inversely related to the development of functional constipation as compared with never breastfed (adjusted OR = 0.76; CI95 %: 0.65–0.88; *p* < 0.001)
Cross-sectional multicenter study in general pediatric clinics Saudi Arabia (Jeddah, Riyadh, Tabouk, Al-Madinah Al-Munawarah, Khamis Mushait, and Dammam) Hasosah (2024) [67]	1011 children aged 0–48 months. Functional constipation prevalence was significantly higher in toddlers aged 13–48 months (34.5 %) compared with infants aged 0–12 months (9.1 %; *p* < 0.001),	Term gestational age infant, partial breastfeeding, formula feeding, and subjects with no history of food allergy are associated with the prevalence of FGIDs. There is no specific, individualized information relating breastfeeding to constipation.
Retrospective–prospective cohort study in a well-baby clinic Songkla, Thailand Chanpong (2025) [68]	All 686 children aged 3 years (20.4 % had functional constipation), who had been followed up with in a well-baby clinic for vaccination since they were 2 months old	Exclusive breastfeeding for ≥ 6 months was a significant protective factor against functional constipation (OR = 0.65, 95 %CI:0.42–0.99, *p* = 0.047).
Hospital-based cross-sectional survey China (14 cities) Wang (2025) [69]	2528 infants aged 0–9 months with GI discomfort (72.6 % fulfilled the criteria for FIGDs) The study does not have a healthy control group	Logistic regression analysis results showed that the duration of exclusive breastfeeding > 4 months (OR = 0.75, 95 % CI: 0.595–0.946; *p* < 0.05) was related to FGIDs

Original articles selected in PubMed (January 2026) using the words “constipation” and “breastfeeding", OR [CI95 %], odds ratio and 95 % confidence interval; FGIDs, functional gastrointestinal disorders.

The first articles were published in Brazil. In 2002, a public primary care-based article from the metropolitan region of São Paulo showed the protective effect of breastfeeding on the development of constipation [35]. The study showed that artificial breastfeeding was associated with a 4.5-fold greater risk of constipation than that of infants who were predominantly breastfed [35]. Another public primary care study also conducted in the metropolitan region of São Paulo showed a 3.0-fold greater risk of constipation in those who received artificial breastfeeding between 6 and 24 months of age [57]. However, a more recent epidemiological study with infants from all regions of Brazil treated in private pediatric practices showed that functional constipation in infants was associated with age and history of prematurity, but not with sex or type of breastfeeding [13]. Whole cow's milk was likely used as a milk source in previous primary care studies [35,51], while in the most recent study [13], in private pediatric practices, >90 % of infants received infant formula as their sole milk source [13,52]. In other words, whole cow's milk may be a risk factor for constipation, which was not observed with the use of infant formula, considering natural breastfeeding as a reference. This hypothesis should be investigated in future studies specifically designed for this purpose.

Table 1 also shows that almost all articles indicate a favorable role for breastfeeding in maintaining normal bowel habits and a lower chance of future constipation. However, it should be noted that there is considerable heterogeneity in the study design, definition of variables, and analytical strategies. Therefore, the specific role of infant formulas with or without added prebiotics and whole cow's milk as risk factors for constipation should be confirmed in future studies specifically designed to answer these questions.

Another interesting observation regarding constipation prevention was obtained from a study conducted in Porto Alegre, Brazil [70]. This randomized cohort study showed that infants cared for in the first two years of life by professionals who had received training in the Ten Steps to Healthy Eating had a 38 % reduced risk of constipation at the age of 6 years [70].

In summary, the premature cessation of breastfeeding is a risk factor for constipation. In this situation, infant formulas should be used, especially those containing prebiotics such as a mixture of fructooligosaccharides (FOSs) and galactooligosaccharides (GOSs), which decrease stool consistency and increase bowel movement frequency [71,72]. There is still very little evidence on the role of synthetic oligosaccharides identical to those found in breast milk (human milk oligosaccharides [HMOs]) added to infant formulas. The results of two clinical trials showed that a dose of 5.8 g/L of HMOs increased bowel movement frequency and decreased stool consistency, a finding not observed in another clinical trial that used infant formula with a lower HMO content [72].

Another concern is the lipid profiles of cow's milk and some infant formulas, which may be associated with the increased formation of calcium soaps with palmitic acid esterified at the sn-1 and sn-2 glycerol positions. Thus, the addition of palmitic acid esterified at the sn-2 position in infant formulas results in better absorption, increases the bioavailability of calcium, and provides stools with less consistency by reducing soponification [73,74].

In summary, the first year of life provides an excellent opportunity for defining food preferences and preventing constipation. In infants, constipation generally begins with pain or difficulty passing hard, separate hard lumps (like nuts) without an increase in the interval between bowel movements [13,35,37]. These clinical manifestations do not meet the Rome IV [8] criteria for functional constipation; however, they serve as warnings that dietary changes should be implemented promptly. After the introduction of complementary foods, dietary fiber can be a key factor in preventing constipation. The addition of prebiotics to infant formula may decrease stool consistency and increase bowel movement frequency compared to formulas without prebiotics and cow's milk, thus reducing the chance of painful bowel movements that can trigger the onset of functional constipation. On the other hand, there is no evidence to justify the recommendation of probiotics in the prevention or treatment of functional constipation according to the ESPGHAN guidelines [75]. The same position is adopted by ESPGHAN for the use of prebiotics in the treatment of functional constipation [76].

### The role of dietary fiber in preventing functional constipation

The benefits of dietary fiber have been recognized since the Hippocratic era; however, to date, there is no fully accepted definition [77., 78., 79., 80., 81., 82., 83., 84.]. Dietary fiber can be defined as polymeric carbohydrates with >10 monosaccharide units that are resistant to enzymatic hydrolysis in the human intestine [77., 78., 79., 80.]. More recently, a broader definition has also been proposed that adopts the term “functional fiber”, which includes oligosaccharides with a lower degree of polymerization that are not absorbed by the intestine and are fermented in the intestinal lumen by the intestinal microbiota [84]. Generally, soluble fibers are more fermentable, have greater osmotic power, and a greater capacity to stimulate the growth of bacterial mass, while insoluble fibers provide an increase in fecal volume/bulk [77., 78., 79., 80., 81., 82., 83., 84.]. Many tables of insoluble and soluble dietary fiber composition in foods are based on the analytical determination recommended by the Association of Official Agricultural Chemists’ methods, which provide the levels of soluble and insoluble fiber [81]. The Dietary Reference Intake (DRI) recommendations include the following definitions: dietary fiber consists of nondigestible carbohydrates and lignin that are intrinsic and intact in plants; functional fiber consists of isolated, nondigestible carbohydrates that have beneficial physiological effects in humans; total fiber is the sum of dietary fiber and functional fiber [84].

For the pediatric age group, there are two commonly used recommendations for dietary fiber intake. In 1995, for children over 2 years of age, the minimum consumption recommended in grams/day was equal to the age expressed in years plus 5, and the maximum was the age plus 10 *g* [85]. This recommendation has been adopted in Brazil, both for research [46,86] and also by the Brazilian Society of Pediatrics [87]. In turn, the DRIs recommend higher values based on the concept of “adequate intake” for total fiber, as shown in Table 2 [84]. It must be noted that there are no dietary fiber recommendations for infants in the first year of life [35,57,84,85].

**Table 2:** Recommendation for dietary fiber intake in pediatrics.

**Fiber definition**	**Recommendations**
Dietary fiber Age + 5 or 10 g Williams et al. 1995	Boys and girls older than 2 years of age: Minimum intake (g/day) = age expressed in years plus 5 Maximum intake (g/day) = age expressed in years plus 10
Adequate intake (AI) for total fiber (dietary fiber + functional fiber) Dietary Reference Intake Institute of Medicine, 2006	Boys and girls: 1–3 years: 19 g/day; 3–4 years: 25 g/day Boys: 9–13 years: 31 g/day; 14–18 years: 38 g/day Girls: 9–13 years: 26 g/day; 14–18 years: 26 g/day

Williams, 1995 [85]; Institute of Medicine, 2006 [84].

Dietary fiber is traditionally recommended as part of the maintenance treatment of constipation, as recommended by the UK National Institute for Health and Care Excellence [22], the World Gastroenterology Organization [23], and also in documents from the Brazilian Society of Pediatrics [80,87]. In this context, in Brazil, since the 1990s, a diet rich in dietary fiber has been considered part of the treatment of constipation [1,45,88]. However, the recommendation of a diet rich in dietary fiber for the treatment of constipation is not based on scientific evidence from clinical trials. Systematic reviews of controlled clinical trials have shown that there is no evidence that dietary fiber contributes to the treatment of constipation [77,79,89]. In this regard, the ESPGHAN, published in 2014, states that a normal dietary fiber intake is recommended in children with constipation [2]. The immediate interpretation of this recommendation is that increasing dietary fiber consumption is not indicated for the treatment of constipation. However, in real-life settings, a large portion of the population, including children and adolescents, consumes insufficient dietary fiber compared to the recommendations, indicating the need to increase dietary fiber consumption in patients with constipation who consume insufficient dietary fiber. In addition, most clinical trials on the efficacy of dietary fiber in the treatment of constipation have been conducted in specialized services where patients with severe constipation are treated. Therefore, the lack of dietary fiber efficacy in patients treated with specialized services should not be extrapolated to the entire population. In specialized services, patients with more severe conditions require a therapeutic program that includes disimpaction and laxatives [2,4., 5., 6.,^19^,^20^], which could make it difficult to demonstrate the specific effects of dietary fiber.

Another interpretation of the relationship between dietary fiber intake and constipation focuses on the possibility that habitually insufficient dietary fiber consumption is a risk factor for constipation. In the late 1990s, a population-based epidemiological study [90] and another case-control study [46] demonstrated for the first time that low dietary fiber consumption is associated with constipation in children and adolescents. In Greece, a negative relationship between dietary fiber consumption and constipation was observed [90]. In Brazil, a case-control study showed an odds ratio of 4.1 linking low dietary fiber consumption and constipation [43]. Other studies have confirmed these findings both in Brazil [91] and in other Eastern countries [92., 93., 94., 95., 96.]. In contrast, other Brazilian studies [86,97., 98., 99.] did not confirm the association between lower dietary fiber consumption and constipation. However, no epidemiological studies have shown an association between excessive dietary fiber intake and an increased risk of constipation.

Interventions, such as dietary fiber and prebiotics, can increase stool frequency and improve fecal consistency in non-breast-fed infants [71,72] and children with isolated symptoms of constipation [100].

Thus, considering the available epidemiological evidence, insufficient fiber consumption is high in the general population and is associated with constipation in several studies. Therefore, a diet that meets the dietary fiber recommendations should be considered to prevent constipation.

### The role of water intake in preventing constipation

Water is essential for life and is the most abundant component of the human body. Water participates in several metabolic processes, including the transport of substances across membranes, cellular homeostasis, temperature regulation, and circulatory physiology. Total body water represents 75 % of an infant's weight and approximately 55 % of an adult’s weight. Water is not stored in the body, and the amount produced during metabolic processes is insufficient to meet the body’s needs. Therefore, it should be consumed throughout the day to ensure adequate hydration. Water needs are influenced by several factors such as age, sex, body mass, physical activity levels, and environmental factors [101., 102., 103.]. In the first months of life, exclusive breastfeeding fully meets water needs [101., 102., 103.]. Table 3 presents the water consumption recommendations according to the Institute of Medicine's DRIs [84]. From the age of 9 years, total water needs are higher for boys. In women, the recommended water intake is higher during pregnancy, particularly during lactation. It should be noted that, from an epidemiological point of view, more than half of the pediatric population does not meet the minimum water intake recommendations [103]. For this reason, hypohydration was also observed in more than half of the children included in studies conducted in Israel, France, the USA, Italy, and Brazil [104,105].

**Table 3:** Recommended daily minimum water consumption (Dietary Reference Intake, DRI).

	Boys (L/day)	Girls (L/day)
0–6 months	0.7	0.7
7–12 months	0.8	0.8
1–3 years	1.3	1.3
4–8 years	1.7	1.7
9–13 years	2.4	2.1
14–18 years	3.3	2.3

Institute of Medicine, 2006 [84].

In this context, the hypothesis that hypohydration is related to the etiology of constipation has been discussed in the literature [106]. In clinical practice, increasing fluid consumption is part of some guidelines for the treatment of constipation [22,23]. However, the NASPGHAN/ESPGHAN guidelines, published in 2014, established, based on expert opinion, that increasing fluid consumption should not be part of the therapeutic program for functional constipation [2].

In turn, a literature review identified only five articles that explored the efficacy of fluid intake in the treatment of constipation [31]. These studies had heterogeneous designs. The results of some studies have suggested a positive effect; however, none of them allowed for an unquestionable position [31]. A significant practical limitation in planning a randomized controlled study on this topic is the difficulty of determining an adequate placebo.

In contrast, only five articles have linked habitual fluid consumption and constipation [31]. All of which showed lower fluid intake among children with constipation, and in three, the difference was statistically significant [31].

The relationship between hypohydration and constipation has been evaluated in only two studies conducted in Brazil [107,108]. The first, a school-based study, evaluated children with and without constipation aged 7–10 years [107]. Children with constipation, compared to the control group, had lower fluid intake and higher mean urinary osmolarity, while the association between hypohydration and constipation did not reach statistical significance (*p* = 0.073) [107]. The second, a case-control study, showed an association between severe constipation and hypohydration in girls in the bivariate analysis. However, statistical significance was not maintained (*p* = 0.082) in the multivariate analysis, including age [108].

Further studies are required to investigate the relationship between hypohydration and constipation. However, available evidence suggests that adequate water intake may protect against the development of constipation.

## Conclusion

The basic principles of constipation treatment are well established; however, the possibility of preventing constipation has rarely been discussed in the literature. Unfortunately, initial clinical manifestations of constipation are often overlooked.

**Figure 1:**
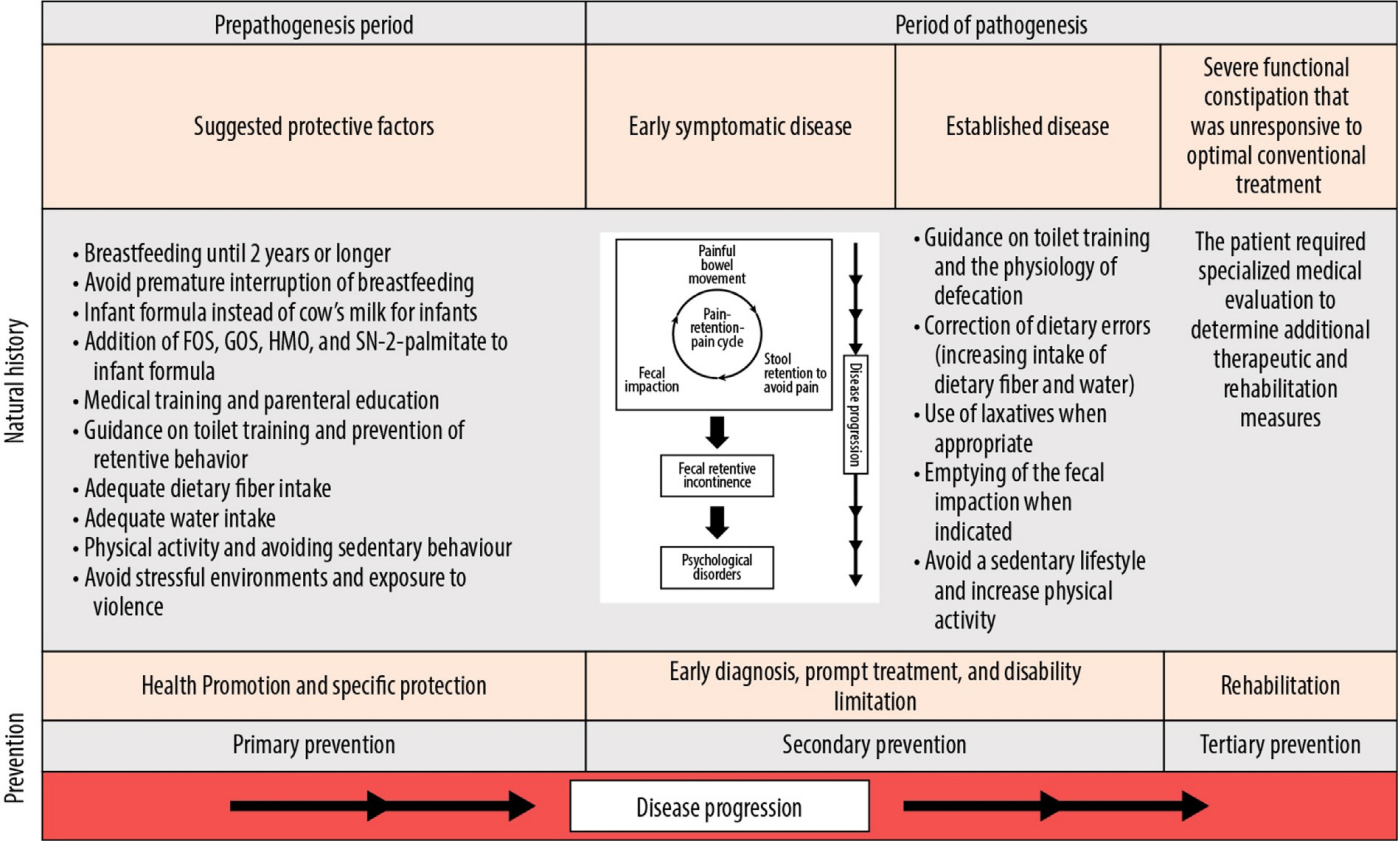
The natural history of functional constipation (adapted from the Leavell and Clark’s model). FOS, fructooligosaccharide; GOS, galactooligosaccharide; HOM, human milk oligosaccharide.

The natural history of functional constipation is shown in Figure 1. Preventive measures should include actions taken before functional constipation reaches the clinical stage and should address the population as a whole.

It should be reaffirmed that the primary prevention measures discussed in this article are important for promoting overall health and should be recommended for all children and adolescents from a holistic perspective.

Furthermore, these preventive measures can be useful in reeducating patients at the end of a successful treatment cycle for functional constipation, aiming to reduce the risk of recurrence of this functional gastrointestinal disorder.

Additionally, avoiding a sedentary lifestyle and encouraging physical activity are recommended to promote health and can be beneficial for bowel function. A recent review found no solid evidence that physical inactivity and sedentary behavior are causally linked to the development of constipation in children [109]. However, the authors highlight the importance of promoting normal physical activity for children with constipation [109].

No prospective population-based studies have evaluated the effectiveness of preventive measures against constipation. However, some measures can contribute to the prevention of functional constipation both individually and from a public health perspective.

In conclusion, the following actions may contribute to the prevention of functional constipation:

• Promotion of exclusive breastfeeding
• When breastfeeding is prematurely discontinued, infant formulas with added prebiotics and sn-2-palmitate should be prioritized over whole-cow milk.
• Avoiding the switching of infant formulas and unnecessary treatments to control physiological gastrointestinal symptoms during the first months of life
• Providing proper toilet training guidance
• Early recognition and correction of fecal retentive behavior.
• Encourage avoiding postponing bowel movements at all stages of life
• Adoption of healthy eating habits, including adequate dietary fiber and fluid intake, is strongly recommended.
• Encourage physical activity and avoid a sedentary lifestyle.

Training healthcare professionals and educating the general population are important for the effective adoption of measures aimed at preventing functional constipation.

## Funding

None.

## Data availability

The data that support the findings of this study are available from the corresponding author.

## Conflicts of interest

The author declares no conflicts of interest.

## References

[1] Morais M.B., Maffei H.V. (2000). Constipação intestinal. J Pediatr.

[2] Tabbers M.M., Dilorenzo C., Berger M.Y., Faure C., Langendam M.W., Nurko S. (2014). Evaluation and treatment of functional constipation in infants and children: evidence-based recommendations from ESPGHAN and NASPGHAN. J Pediatr Gastroenterol Nutr.

[3] Rajindrajith S., Devanarayana N.M., Perera B.J., Benninga M.A. (2016). Childhood constipation as an emerging public health problem. World J Gastroenterol.

[4] Vriesman M.H., Koppen I.J., Camilleri M., Di L.C., Benninga M.A. (2020). Management of functional constipation in children and adults. Nat Rev Gastroenterol Hepatol.

[5] Morais M.B., Barueri M. (2022). Tratado de pediatria SBP. 5ª.

[6] Tran D.L., Sintusek P. (2023). Functional constipation in children: what physicians should know. World J Gastroenterol.

[7] Vandenplas Y., Hauser B., Salvatore S. (2019). Functional gastrointestinal disorders in infancy: impact on the health of the infant and family. Pediatr Gastroenterol Hepatol Nutr.

[8] Benninga M.A., Nurko S., Faure C., Hyman P.E., St J., Roberts I., Schechter N.L. (2016). Childhood functional gastrointestinal disorders: neonate/toddler. Gastroenterology.

[9] Vandenplas Y., Broekaert I., Domellöf M., Indrio F., Lapillonne A., Pienar C. (2024). An ESPGHAN position paper on the diagnosis, management, and prevention of cow's milk allergy. J Pediatr Gastroenterol Nutr.

[10] de Oliveira L.C., Silva L.R., Franco J.M., Watanabe A.S., Pinto-Júnior A.B., Capelo A. (2025). Atualização em alergia alimentar 2025: posicionamento conjunto da Associação Brasileira de Alergia e Imunologia e Sociedade Brasileira de Pediatria. Arq Asma Alerg Imunol.

[11] Hyams J.S., Di Lorenzo C., Saps M., Shulman R.J., Staiano A., Van Tilburg M. (2016). Childhood functional gastrointestinal disorders: child/adolescent. Gastroenterology.

[12] Steutel N.F., Zeevenhooven J., Scarpato E., Vandenplas Y., Tabbers M.M., Staiano A. (2020). Prevalence of functional gastrointestinal disorders in European infants and toddlers. J Pediatr.

[13] Morais M.B., Toporovski M.S., Tofoli M.H., Barros K.V., Silva L.R., Ferreira C.H. (2022). Prevalence of functional gastrointestinal disorders in Brazilian infants seen in private pediatric practices and their associated factors. J Pediatr Gastroenterol Nutr.

[14] Velasco-Benítez C.A., Collazos-Saa L.I., García-Perdomo H.A. (2022). A systematic review and meta-analysis in schoolchildren and adolescents with functional gastrointestinal disorders according to Rome iv criteria. Arq Gastroenterol.

[15] Sperber A.D., Bangdiwala S.I., Drossman D.A., Ghoshal U.C., Simren M., Tack J. (2021). Worldwide prevalence and burden of functional gastrointestinal disorders. Gastroenterology.

[16] Maffei H.V., de Morais M.B. (2005). Defining constipation in childhood and adolescence: from Rome, via Boston, to Paris and …?. J Pediatr Gastroenterol Nutr.

[17] Maffei H.V., Morais M.B. (2018). Proposals to approximate the pediatric Rome constipation criteria to everyday practice. Arq Gastroenterol.

[18] Silva L.B., Dias F.C., Melli L.C., Tahan S., Morais M.B. (2022). Clinical spectrum of functional constipation and bowel-habit patterns of schoolchildren recruited from two elementary schools and a specialized outpatient clinic. Arq Gastroenterol.

[19] Loening-Baucke V. (1993). Chronic constipation in children. Gastroenterology.

[20] Baker S.S., Liptak G.S., Colletti R.B., Croffie J.M., Di Lorenzo C., Ector W. (1999). Constipation in infants and children: evaluation and treatment. A medical position statement of the North American Society for Pediatric Gastroenterology and Nutrition. J Pediatr Gastroenterol Nutr.

[21] Constipation Guideline Committee of the North American Society for Pediatric Gastroenterology, Hepatology and Nutrition (2006). Evaluation and treatment of constipation in infants and children: recommendations of the North American Society for Pediatric Gastroenterology, Hepatology and Nutrition. J Pediatr Gastroenterol Nutr.

[22] National Collaborating Centre for Women's and Children's Health (UK) (2010).

[23] Lindberg G., Hamid S.S., Malfertheiner P., Thomsen O.O., Fernandez B., Garisch J.J. (2011). World gastroenterology organization global guideline: constipation - A global perspective. J Clin Gastroenterol.

[24] Kilgore A.L., Rogers Boruta M.K., Ambartsumyan L., Suarez R.G., Patel D., Wood R.J. (2025). Evaluation and management of pediatric refractory constipation: recommendations from the NASPGHAN neurogastroenterology and motility committee. J Pediatr Gastroenterol Nutr.

[25] Sinopoulou V., Gordon M., Rajindrajith S., Hathagoda W., Rane A.B., Sedghi A. (2024). How do we define therapy-resistant constipation in children aged 4-18 years old? A systematic review with meta-narrative synthesis. BMJ Paediatr Open.

[26] Rappaport L.A., Levine M.D. (1986). The prevention of constipation and encopresis: a developmental model and approach. Pediatr Clin North Am.

[27] Rogers J. (2012). Assessment, prevention and treatment of constipation in children. Nurs Stand.

[28] Indrio F., Di Mauro A., Riezzo G., Civardi E., Intini C., Corvaglia L. (2014). Prophylactic use of a probiotic in the prevention of colic, regurgitation, and functional constipation: a randomized clinical trial. JAMA Pediatr.

[29] Motaharifard M.S., Jafari Z., Paknejad M.S., Oveidzadeh L., Karimi M. (2016). Prevention and treatment of constipation in children from the perspective of Iranian traditional medicine. J Integr Med.

[30] Ferrara L.R., Saccomano S.J. (2017). Constipation in children: diagnosis, treatment, and prevention. Nurse Pr.

[31] Boilesen S.N., Tahan S., Dias F.C., Melli L.C., de Morais M.B. (2017). Water and fluid intake in the prevention and treatment of functional constipation in children and adolescents: is there evidence?. J Pediatr Rio J.

[32] den Hertog J., van Leengoed E., Kolk F., van den Broek L., Kramer E., Bakker E.J. (2012). The defecation pattern of healthy term infants up to the age of 3 months. Arch Dis Child Fetal Neonatal ed.

[33] Weaver L.T., Ewing G., Taylor L.C. (1988). The bowel habit of milk-fed infants. J Pediatr Gastroenterol Nutr.

[34] Gatzinsky C., Sillén U., Thornberg S., Sjöström S. (2023). Bowel habits in healthy infants and the prevalence of functional constipation, infant colic and infant dyschezia. Acta Paediatr.

[35] Aguirre A.N., de C., Vitolo M.R., Puccini R.F., Morais M.B. (2002). Constipação em lactentes: influência do tipo de aleitamento e da ingestão de fibra alimentar. J Pediatr Rio J.

[36] Sociedade Brasileira de Pediatria e Sociedade Brasileira de Urologia. Manual de Orientação: Treinamento esfincteriano. 2019. 11p

[37] Medeiros L.C., Morais M.B., Tahan S., Fukushima E., Motta M.E. (2007). Fagundes-Neto U. Características clínicas de pacientes pediátricos com constipação crônica de acordo com o grupo etário. Arq Gastroenterol.

[38] Thomaz de Almeida C.N., Tahan S., Areco K.N., Morais M.B (2021). Association between abuse and neglect with functional constipation and irritable bowel syndrome in adolescents. Scand J Gastroenterol.

[39] Carneiro J.F., Silva E.P., da Silva G.A., Ludermir A.B. (2023). Could children exposed to intimate partner violence against their mother have more functional gastrointestinal disorders?. J Pediatr Rio J.

[40] Mello C.S., Freitas K.C., Tahan S., Morais M.B. (2010). Dietary fiber intake for children and adolescents with chronic constipation: influence of mother or caretaker and relationship with overweight. Rev Paul Pediatr.

[41] Peeters B., Benninga M.A., Hennekam R.C. (2011). Childhood constipation; an overview of genetic studies and associated syndromes. Best Pr Res Clin Gastroenterol.

[42] Tappin D., Grzeda M., Joinson C., Heron J. (2020). Challenging the view that lack of fibre causes childhood constipation. Arch Dis Child.

[43] Goshima S., Fagundes-Neto U., Morais M.B. (2004). Dermatóglifos em crianças com constipação crônica. Arq Gastroenterol.

[44] Oliveira J.N., Tahan S., Goshima S., Fagundes-Neto U., Morais M.B. (2006). Prevalência de constipação em adolescentes matriculados em escolas de São José dos Campos, SP, e em seus pais. Arq Gastroenterol.

[45] Moreira F.L., Kissimoto M., Chaves S.M.F., El Faro S., Aleixo A.M. (1994). História clínica e alimentar de crianças atendidas em ambulatório de gastroenterologia pediátrica com constipação intestinal crônica funcional e suas possíveis complicações. J Pediatr Rio J.

[46] Morais M.B., Vítolo M.R., Aguirre A.N., Fagundes-Neto U. (1999). Measurement of low dietary fiber intake as a risk factor for chronic constipation in children. J Pediatr Gastroenterol Nutr.

[47] Koppen I.J.N., Vriesman M.H., Saps M., Rajindrajith S., Shi X., van Etten-Jamaludin F.S. (2018). Prevalence of functional defecation disorders in children: a systematic review and meta-analysis. J Pediatr.

[48] Morais M.B. (2016). Signs and symptoms associated with digestive tract development. J Pediatr Rio J.

[49] Victora C.G., Bahl R., Barros A.J., França G.V., Horton S., Krasevec J. (2016). Breastfeeding in the 21st century: epidemiology, mechanisms, and lifelong effect. Lancet.

[50] Boccolini C.S., Boccolini P.M., Monteiro F.R., Venancio S.I., Giugliane E.R. (2017). Tendência de indicadores do aleitamento materno no Brasil em três décadas. Rev Saude Publica.

[51] Morais M.B., Cardoso A.L., Lazarini T., Mosquera E.M., Mallozi M.C (2017). Habits and attitudes of mothers of infants in relation to breastfeeding and artificial feeding in 11 Brazilian cities. Rev Paul Pediatr.

[52] Morais M.B., Toporovski M.S., Tofoli M.H., Barros K.V., Ferreira C.H., Silva L.R. (2022). Breastfeeding in infants seen in private pediatric practices and its relation with type of delivery and history of prematurity. J Pediatr Rio J.

[53] IBFAN, WHO (2020).

[54] de Morais M.B., Vítolo M.R., Aguirre A.N., Medeiros E.H., Antoneli E.M. (1996). Fagundes-Neto U. Intake of dietary fiber and other nutrients by children with and without functional chronic constipation. Arq Gastroenterol.

[55] Iacono G., Merolla R., D'Amico D., Bonci E., Cavataio F., Di Prima L. (2005). Gastrointestinal symptoms in infancy: a population-based prospective study. Dig Liver Dis.

[56] Tunc V.T., Camurdan A.D., Ilhan M.N., Sahin F., Beyazova U. (2008). Factors associated with defecation patterns in 0-24-month-old children. Eur J Pediatr.

[57] Souza D.S., Tahan S., Morais M.B. (2012). Constipation and dietary fiber intake in infants: relation with type of feeding, nutritional status and indicators of body iron. Rev Med Minas Gerais.

[58] Çamurdan A.D., Beyazova U., Özkan S., Tunç V.T. (2014). Defecation patterns of the infants mainly breastfed from birth till the 12th month: prospective cohort study. Turk J Gastroenterol.

[59] Park M., Bang Y.G., Cho K.Y. (2016). Risk factors for functional constipation in young children attending daycare centers. J Korean Med Sci.

[60] Heron J., Grzeda M., Tappin D., von Gontard A., Joinson C. (2018). Early childhood risk factors for constipation and soiling at school age: an observational cohort study. BMJ Paediatr Open.

[61] Pattison K.L., Kraschnewski J.L., Lehman E., Savage J.S., Downs D.S., Leonard K.S. (2019). Breastfeeding initiation and duration and child health outcomes in the first baby study. Prev Med.

[62] Chew K.S., Em J.M., Koay Z.L., Jalaludin M.Y., Ng R.T., Lum L.C. (2021). Low prevalence of infantile functional gastrointestinal disorders (FGIDs) in a multi-ethnic Asian population. Pediatr Neonatol.

[63] Agakisiyeva G., Yildirim D., Hizarcioglu-Gulsen H., Gumus E., Karhan A.N., Karabulut E. (2022). Nutritional characteristics of patients with functional constipation aged 4 years and older. Minerva Pediatr.

[64] de Oliveira M.B., Jardim-Botelho A., de Morais M.B., da Cruz Melo I.R., Maciel J.F., Gurgel R.Q. (2021). Impact of infant milk-type and childhood eating behaviors on functional constipation in preschool children. J Pediatr Gastroenterol Nutr.

[65] Chia L.W., Nguyen T.V., Phan V.N., Luu T.T., Nguyen G.K., Tan S.Y. (2022). Prevalence and risk factors of functional gastrointestinal disorders in Vietnamese infants and young children. BMC Pediatr.

[66] Motoki N., Inaba Y., Toubou H., Hasegawa K., Shibazaki T., Tsukahara T. (2023). Japan Environment and Children’s Study (JECS) Group. Impact of breastfeeding during infancy on functional constipation at 3 years of age: the Japan Environment and Children's Study. Int Breastfeed J.

[67] Hasosah M., Al Sarkhy A., AlQuiar K., AlMuslami I., AlAhmadi N., Almehaidib A. (2024). Prevalence of functional gastrointestinal disorders in Saudi infants and toddlers: a cross-sectional multicenter study. Saudi J Gastroenterol.

[68] Chanpong A., Ponjorn N., Tassanakijpanich N., Koosakulchai V., Rachatawiriyakul P., Kittivisuit S. (2025). Early-life events and the prevalence of gut-brain interaction disorders in children. Child.

[69] Wang Z., Jia X., Hu C., Wu J., Hu D., Zhong Y. (2025). Prevalence and influencing factors of functional gastrointestinal disorders among infants aged 0 to 9 months with gastrointestinal discomfort in China: a multicenter cross-sectional survey. Sichuan Xue Xue Bao Yi Xue Ban.

[70] Sangalli C.N., Leffa P.D., Morais M.B., Vitolo M.R. (2018). Infant feeding practices and the effect in reducing functional constipation 6 years later: a randomized field trial. J Pediatr Gastroenterol Nutr.

[71] Moro G.E., Mosca F., Miniello V., Fanaro S., Jelinek J., Stahl B. (2003). Effects of a new mixture of prebiotics on faecal flora and stools in term infants. Acta Paediatr Suppl.

[72] Leong A., Mori C., Pillidge C., Gill H. (2024). Do oligosaccharide-supplemented infant formulas improve infant gastrointestinal health? A systematic review of randomized clinical trials. Food Rev Int.

[73] Bronsky J., Campoy C., Embleton N., Fewtrell M., Mis N.F., Gerasimidis K. (2019). Palm oil and beta-palmitate in infant formula: a position paper by the European Society for Paediatric Gastroenterology, Hepatology, and Nutrition (ESPGHAN) Committee on Nutrition. J Pediatr Gastroenterol Nutr.

[74] Sheng X.Y., Mi W., Yuan Q.B., Liu B.Y., Carnielli V., Ning Y.B. (2024). An A2 β-casein infant formula with high sn-2 palmitate and casein phosphopeptides supports adequate growth, improved stool consistency, and bone strength in healthy, term Chinese infants: a randomized, double-blind, controlled clinical trial. Front Nutr.

[75] Szajewska H., Berni Canani R., Domellöf M., Guarino A., Hojsak I., Indrio F. (2023). Probiotics for the management of pediatric gastrointestinal disorders: position paper of the ESPGHAN Special Interest Group on gut microbiota and modifications. J Pediatr Gastroenterol Nutr.

[76] Indrio F., Dinleyici E.C., Berni Canani R., Domellöf M., Francavilla R., Guarino A. (2024). Prebiotics in the management of pediatric gastrointestinal disorders: position paper of the ESPGHAN special interest group on gut microbiota and modifications. J Pediatr Gastroenterol Nutr.

[77] Hojsak I., Benninga M.A., Hauser B., Kansu A., Kelly V.B., Stephen A.M. (2022). Benefits of dietary fibre for children in health and disease. Arch Dis Chil.

[78] Feng Y., Jin Q., Liu X., Lin T., Johnson A., Huang H. (2025). Advances in understanding dietary fiber: classification, structural characterization, modification, and gut microbiome interactions. Compr Rev Food Sci Food Saf.

[79] Salvatore S., Battigaglia M.S., Murone E., Dozio E., Pensabene L., Agosti M. (2023). Dietary fibers in healthy children and in pediatric gastrointestinal disorders: a practical guide. Nutrients.

[80] Morais M.B., Weffort V.R., Mello E.D. (2024). Manejo nutrológico da constipação intestinal funcional. Manual de Aspectos nutricionais em situações especiais na infância e adolescência. Sociedade Brasileira de Pediatria. - São Paulo: SBP.

[81] Shils M., Olson S., Shike M. (1994).

[82] Burkitt D.P. (1969). Related disease–related cause?. Lancet.

[83] Trowell H., Southgate D.A., Wolever T.M., Leeds A.R., Gassull M.A., Jenkins D.J. (1976). Letter: dietary fibre redefined. Lancet.

[84] Institute of Medicine (2006).

[85] Williams C.L., Bollella M., Wynder E.L. (1995). A new recommendation for dietary fiber in childhood. Pediatrics.

[86] de Carvalho E.B., Vitolo M.R., Gama C.M., Lopez F.A., Taddei J.A., de Morais M.B. (2006). Fiber intake, constipation, and overweight among adolescents living in Sao Paulo City. Nutrition.

[87] Weffort V.R. Manejo nutricional na obstipação intestinal. In: Weffort vrs & lamounier JA. nutrição em pediatria: da neonatologia a adolescencia. 2a editor Manole: Barueri; 2017.

[88] Maffei H.V., Vicentini A.P. (2011). Prospective evaluation of dietary treatment in childhood constipation: high dietary fiber and wheat bran intake are associated with constipation amelioration. J Pediatr Gastroenterol Nutr.

[89] Piccoli de Mello P., Eifer D.A., Daniel de Mello E. (2018). Use of fibers in childhood constipation treatment: systematic review with meta-analysis. J Pediatr Rio J.

[90] Roma E., Adamidis D., Nikolara R., Constantopoulos A., Messaritakis J. (1999). Diet and chronic constipation in children: the role of fiber. J Pediatr Gastroenterol Nutr.

[91] Gomes R.C., Maranhão H.S., Pedrosa L.F., Morais M.B. (2003). Consumo de fibra alimentar e de macronutrientes por crianças com constipação crônica funcional. Arq Gastroenterol.

[92] Ip K.S., Lee W.T., Chan J.S., Young B.W (2005). A community-based study of the prevalence of constipation in young children and the role of dietary fibre. Hong Kong Med J.

[93] Chan M.F., Chan Y.L. (2010). Investigating factors associated with functional constipation of primary school children in Hong Kong. J Clin Nurs.

[94] Chien L.Y., Liou Y.M., Chang P. (2011). Low defaecation frequency in Taiwanese adolescents: association with dietary intake, physical activity and sedentary behaviour. J Paediatr Child Health.

[95] Asakura K., Masayasu S., Sasaki S. (2017). Dietary intake, physical activity, and time management are associated with constipation in preschool children in Japan. Asia Pac J Clin Nutr.

[96] Okuda M., Kunitsugu I., Yoshitake N., Sasaki S. (2019). The relationship between functional constipation and dietary habits in school-age Japanese children. J Nutr Sci Vitaminol.

[97] Dias F.C., Boilesen S.N., Tahan S., Melli L., Morais M.B. (2023). Overweight status, abdominal circumference, physical activity, and functional constipation in children. Rev Assoc Med Bras.

[98] Andreoli C.S., Vieira-Ribeiro S.A., Fonseca P.C., Moreira A.V., Ribeiro S.M., Morais M.B. (2019). Eating habits, lifestyle and intestinal constipation in children aged four to seven years. Nutr Hosp.

[99] Macêdo M.I., Albuquerque M.F., Tahan S., Morais M.B. (2020). Is there any association between overweight, physical activity, fat and fiber intake with functional constipation in adolescents?. Scand J Gastroenterol.

[100] Toporovski M.S., de Morais M.B., Abuhab A., Crippa Júnior M.A (2021). Effect of polydextrose/fructooligosaccharide mixture on constipation symptoms in children aged 4 to 8 years. Nutrients.

[101] Armstrong L.E., Johnson E.C. (2018). Water intake, water balance, and the elusive daily water requirement. Nutrients.

[102] Chouraqui J.P., Thornton S.N., Seconda L., Kavouras S.A. (2022). Total water intake and its contributors in infants and young children. Br J Nutr.

[103] Suh H., Kavouras S.A. (2019). Water intake and hydration state in children. Eur J Nutr.

[104] Dias F.C., Boilesen S.N., Tahan S., Melli L.C., Morais M.B. (2019). Prevalence of voluntary dehydration according to urine osmolarity in elementary school students in the metropolitan region of São Paulo, Brazil. Clin.

[105] Clayton P., Trak-Fellermeier M.A., Macchi A., Galván R., Bursac Z., Huffman-Ercanli F. (2023). The association between hydration status and total fluid intake in healthy children and adolescents. Pediatr Res.

[106] Arnaud M.J. (2003). Mild dehydration: a risk factor of constipation?. Eur J Clin Nutr.

[107] Boilesen S.N., Dias F.C., Tahan S., Melli L.C., de Morais M.B. (2021). Fluid intake and urinary osmolality in pediatric patients with functional constipation. Eur J Nutr.

[108] Dias F.C., Melli L.C., Boilesen S.N., Tahan S., Morais M.B. (2023). Hypohydration, functional constipation, and physical activity in elementary school students. J Pediatr Gastroenterol Nutr.

[109] Adil S., Gordon M., Hathagoda W., Kuruppu C., Benninga M.A., Rajindrajith S. (2024). Impact of physical inactivity and sedentary behaviour on functional constipation in children and adolescents: a systematic review. BMJ Paediatr Open.

